# A Card

**Published:** 1891-03

**Authors:** F. E. Daniel

**Affiliations:** Austin, Texas


### A CARD.

The exposure of the "Hepatic Pill" enterprise, in our December number, has precipitated upon us an avalanche of personal abuse and invective; it has brought the whole pack, "Blanche, Tray and Sweetheart," to snapping at our heels.

By certain gentlemen, for some of whom we have a high regard, we have been accused of an "unwarranted attack upon Dr. Ghent." Nothing could be more absurd. It was an attack, if attack at all, upon a *principle*, a specific act; and a very questionable one, to say the least of it; an act which, had it been committed by a friendless man, would have been universally condemned. Witness the expulsion (in '87) of a younger, and therefore, presumably, a less discreet member, for a very similar, though less flagrant act.

The exposure was, moreover, in the line of duty, and in pursuance of the declared aims, objects and mission of the JOURNAL, the advocacy of legitimate medicine and a *clean profession*. Let those who censure us reflect for a moment, and ask themselves if they are not defending the *man*, and not the act? If so, is one man's character any more sacred than that of another, and can this man do no wrong? (See Transactions 1887, proceedings.)

Does any man who values his own professional character, or who has any regard for the dignity of medicine, pretend to say that such practice is defensible in *any* man? Has the Doctor ever denied knowledge of the sale of the pills so labelled? No where in his alleged "explanation" does he say he did not know it; *he cannot deny it;* we challenge him on his honor to deny it. And, knowing it, what was his plain duty? To stop it. Did he do it? Not until attention was called to it in the JOURNAL. The druggist cannot screen him by saying he had the labels printed without his knowledge. That may be; yet he does not say Dr. Ghent did not become aware of it, or rebuke him for such liberty, and order it stopped and the labels destroyed.

But supposing the editorial did have an element of personality, as alleged; let them ask any member who was at Fort Worth to explain the conduct of the gentleman—now posing as a martyr --conduct with reference to myself; ask them who has been the aggressor in this matter?

I cannot, in justice to my subscribers, who pay for the JOURNAL and are not interested in our personal quarrels, take up its space in personal controversy; but let my friends have no fears but that I will in due time and in the proper manner defend both my position and myself; nor that I will fail at the same time to substantiate every assertion I have made, as well as refute the slanders which have been heaped upon me from another quarter.

Respectfully,

F. E. DANIEL.

Austin, Texas, March 17, 1891.

